# Risk factors for death in children with critical and severe hand-foot-and-mouth disease in Chongqing, China: An observational study

**DOI:** 10.1097/MD.0000000000008934

**Published:** 2017-12-08

**Authors:** Gaihuan Zheng, Jiaoyang Cao, Jie Yu, Zhenzhen Zhang, Quanbo Liu, Junhua Chen

**Affiliations:** Infection Department of the Children's Hospital, The Pediatrics Institution of Chongqing Medical University, Ministry of Education Key Laboratory of Child Development and Disorders, Key Laboratory of Pediatrics in Chongqing, The Children's Hospital of Chongqing Medical University, Chongqing, P.R. China.

**Keywords:** children, death, hand-foot-and-mouth disease, risk factor

## Abstract

Hand-foot-and-mouth disease (HFMD) is a common childhood infection that may lead to serious complications and even death. Globally, epidemics of HFMD are increasing each year, especially in China. This study aimed to identify risk factors for death in children with critical and severe HFMD in Chongqing, China.

We performed an observational study involving patients with critical and severe HFMD admitted to the Children's Hospital of Chongqing Medical University from January 2009 to December 2016. Overall, 179 patients aged 2 months to 16 years, were included; 127 died (non-survival group) and 52 survived (survival group); the case-fatality rate was 70.94%. Data comprising demographic characteristics, clinical symptoms and signs, and laboratory findings were collected. Non-conditional logistic regression analysis was performed to determine the risk factors for death.

Univariate analysis showed that sex, coma, light-reflex insensitivity, pulmonary rales, pulmonary edema or hemorrhage, cold extremities, tachycardia, hypotension, white blood cell count, blood glucose concentration, serum lactate level, creatine kinase-MB isoenzyme level, and acidosis were associated with death (*P* *<* .05). Logistic regression analysis identified female sex (odds ratio [OR] 9.6, 95% confidence interval [CI] 3.0–30.2), light-reflex insensitivity (OR 4.4, 95% CI 1.4–13.1), tachycardia (OR 1.05, 95% CI 1.03–1.07), and higher serum lactate levels (OR 1.14, 95% CI 1.19–1.69) as independent risk factors; and longer onset-to-hospitalization time (OR 0.43, 95% CI 0.28–0.66) as an independent protective factor for death in children with critical and severe HFMD.

Female sex, light-reflex insensitivity, tachycardia, and higher serum lactate level are potential independent risk factors; and longer onset-to-hospitalization time is possibly an independent protective factor for death in patients with critical and severe HFMD.

## 1 Introduction

Hand-foot-and-mouth disease (HFMD) is a common childhood infection that is typically characterized by fever, mouth ulcers, and a papulovesicular rash involving the hands, soles of the feet, and/or buttocks.^[1]^ It is caused by a group of enteroviruses, most commonly, coxsackievirus 16 (CA16) and enterovirus 71 (EV71).^[2,3]^ In recent years, China has been a major region for HFMD outbreaks, particularly due to EV71.^[4,5]^ Although the majority of HFMD cases are mild and self-limiting, severe complications such as encephalitis, meningitis, acute flaccid paralysis, myocarditis, and pulmonary edema have been reported.^[5,6]^ The incidence of severe cases is not rare, especially in China. In severe cases, complications may lead to serious sequelae or death.^[5,7]^ Studies, largely from China, have been performed to identify risk factors associated with severe HFMD.^[8–13]^ However, there is a paucity of data on the risk factors associated with mortality in children with critical and severe HFMD. Thus, discovering relevant early clinical and/or laboratory examination indexes to identify cases at risk of death would be an effective way to support early medical intervention and reduce mortality.

## 2 Methods

For the purpose of this study, HFMD was clinically defined as the presence of oral ulcers and a papulovesicular rash involving the hands, feet, and/or buttocks, with an acute prodromal fever. Patients who had a rash, with or without fever, and no other organ damage, were classified under common HFMD; those who had clinical manifestations involving the nervous system, such as drowsiness, startle, delirium, headache, vomiting, limb-shaking, and ataxia, were classified under generally severe HFMD. Children with HFMD disease accompanied by any of the following: frequent twitching, coma, cerebral hernia, dyspnea, cyanosis, pulmonary hemorrhage, pulmonary edema, shock, and circulatory failure, were classified under critical and severe HFMD.^[14]^ Only critical and severe HFMD cases were incorporated into this study. Patients who either had direct physical contact with children diagnosed with HFMD or had suspected indirect contact, for example, attending the same kindergarten or living in the same quarters as a child with HFMD, within 10 days, were considered to have contact history. Informed consent was received from the patient's family members and the study was approved by the ethics committee.

### 2.1 Study subjects

This retrospective study included 179 patients, classified as having critical and severe HFMD and 2 months to 16 years old, who were admitted to Chongqing Medical University Children's Hospital from January 2009 to December 2016. None of the patients had a congenital disease or had been born prematurely. The cases included 127 deaths (non-survival group) and 52 survivals (survival group), and the case fatality rate was 70.94%.

### 2.2 Data source

Data on demographic characteristics, clinical symptoms and signs, and laboratory findings were collected through a review of medical records. The patient characteristics included age, sex, contact history, onset-to-hospitalization (OH) time, birth weight, breastfeeding, and whether they belonged to the floating population. The clinical symptoms and signs included the recorded peak body temperature, distribution of rash, coma, light-reflex insensitivity, vomiting, myoclonic jerks, headache, pulmonary rales, pneumonedema or pneumorrhagia, tachycardia, and hypoperfusion (as indicated by cold extremities, hypotension, and cyanosis). Laboratory findings included total white blood cell (WBC) count, platelet count, blood glucose concentration, serum lactate concentration, creatine kinase-MB (CK-MB) level, alanine transaminase (ALT) level, arterial blood gas analysis, serum electrolyte levels, and fecal examination for pathogens (EV71, CA16, and enterovirus universal).

### 2.3 Statistical analysis

Continuous variables are presented as mean ± standard deviation, and categorical variables as absolute numbers and proportions. Continuous data were analyzed using Student *t* test. The Kruskal-Wallis test was used for ordered categorical data or non-normally distributed data. Categorical data were tested using the chi-square test or Fisher exact test, as appropriate. We performed a logistic regression analysis (stepwise selection; significance levels to enter and remain in the model were .05 and .10, respectively) to identify the predictors that were discriminatory for death using variables with a significance of <.05 in the univariate analysis. All analyses were performed using SAS 9.2 TS Level 2M3 (Copyright © 2002–2008 by SAS Institute Inc, Cary, NC). A *P* value <.05 was considered statistically significant.

## 3 Results

### 3.1 General characteristics

A total of 112 boys and 67 girls were included in the study. The mean age of the cohort was 2.17 ± 1.19 years. The mean OH time was 3.2 ± 1.26 days (male vs female: 3.11 ± 1.31 vs 3.37 ± 1.16, *P* = .17). There were no statistically significant differences in birth weight, breastfeeding rate, and population mobility between the 2 groups (Table 1).

**Table 1:**
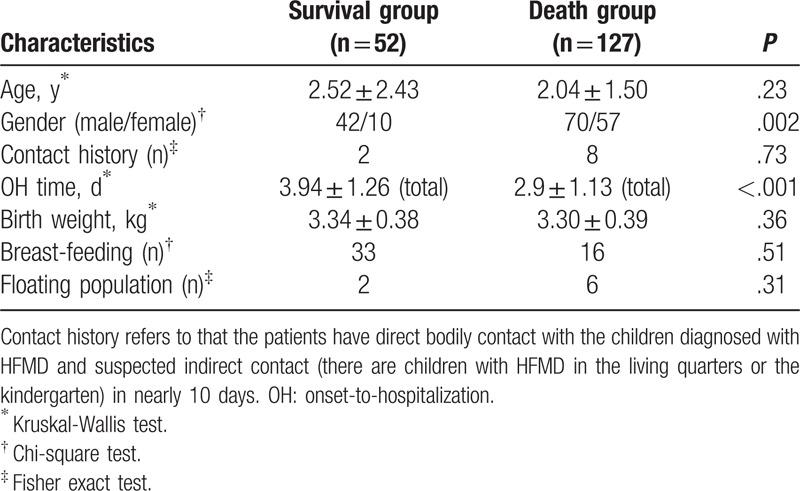
Patients’ general characteristics.

### 3.2 Univariate analysis of death risk factors

The patients’ demographic data are summarized in Table 1. A significant difference was observed in the sex ratio (male/female, 42/10 vs 70/57, *P* = .001) and OH time (3.94 ± 1.26 days, vs 2.9 ± 1.13 days, *P* < .001) between the survival and non-survival groups. A positive contact history (*P* = .73), age (*P* = .23), birth weight (*P* = .36), breastfeeding (*P* = .51), and floating population (*P* = .31) did not predict death. As shown in Table 2, children in the non-survival group had a significantly higher incidence of coma (67.3% vs 89.0%, *P* < 0.001), light-reflex insensitivity (65.4% vs 92.9%, *P* < .001), pulmonary rales (82.6% vs 93.7%, *P* = .02), pulmonary edema or hemorrhage (42.3% vs 59.1%, *P* < .001), cold extremities (hypoperfusion) (57.7% vs 92.9%, *P* < .001), tachycardia (182.4 ± 24.0 beats/min vs 206.05 ± 22.5 beats/min, *P* < .001), and hypotension (23.1% vs 65.4%, *P* < .001). However, there were no significant differences in the peak body temperature (*P* = .28) or the occurrence of atypical rashes (*P* = .90), vomiting (*P* = .48), myoclonic jerks (*P* = .08), headache (*P* = .33), and cyanosis (*P* = .07).

**Table 2:**
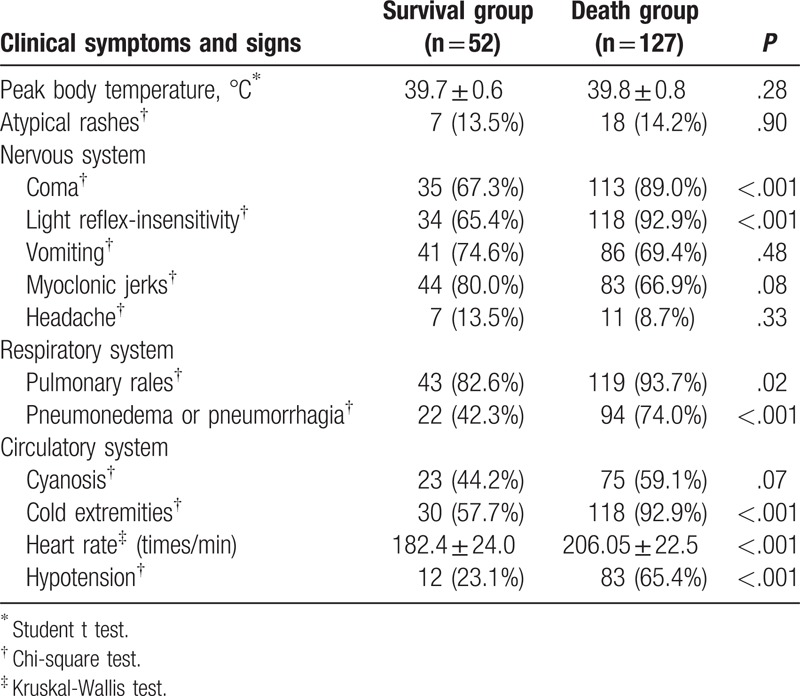
Clinical symptoms and signs.

As shown in Table 3, death was associated with a higher WBC count (13.7 ± 4.9 × 10^9^ L^−1^ vs 18.2 ± 7.7 × 10^9^ L^−1^, *P* *<* .001), blood glucose concentration (10.3 ± 5.2 mmol/L vs 15.3 ± 7.3 mmol/L, *P* *<* .001), serum lactate level (2.9 ± 2.3 vs 6.4 ± 4.0 mmol/L, *P* < .001), CK-MB level (33.2 ± 35.4 U/L vs 46.7 ± 48.7 U/L, *P* = .03), and acidosis (30.8% vs 59.8%, *P* < .001). However, there were no statistically significant differences in platelet count (*P* = .99), ALT level (*P* = .43), and incidence of electrolyte disturbance (*P* = .42) between the 2 groups. Moreover, the fecal samples of 124 patients (52 in the survival group and 72 in the non-survival group) underwent virology examination; EV71 positivity was not significantly associated with the risk of fatal HFMD (*P* = .66).

**Table 3:**
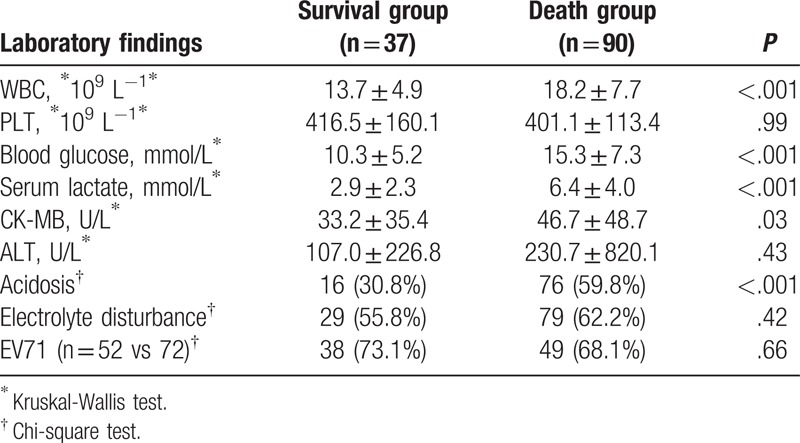
Laboratory findings.

### 3.3 Logistic regression analysis of risk factors for death

To adjust for confounding factors, logistic regression was used. Variables that were statistically significant in the univariate analysis (sex, OH time, coma, light-reflex insensitivity, pulmonary rales, pulmonary edema or hemorrhage, cold extremities, heart rate, hypotension, WBC count, blood glucose concentration, serum lactate level, CK-MB level, and acidosis) were included in the multivariate analysis. Female sex (OR 17.38, 95% CI 4.57–66.12), light reflex insensitivity (OR 3.78, 95% CI 1.18–12.16), tachycardia (OR 1.06, 95% CI 1.03–1.08), and serum lactate levels (OR 1.36, 95% CI 1.13–1.63) were identified as independent risk factors; and longer OH time (OR 0.43, 95% CI: 0.28–0.66) was identified as an independent protective factor for death in children with critical and severe HFMD. At the same time, no interaction effect was found between gender and OH time (*P* = .07) (Table 4).

**Table 4:**
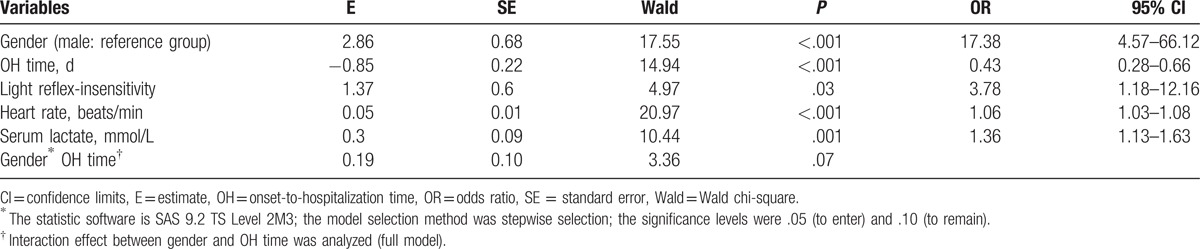
Logistic regression analysis of risk factors for death^∗^.

## 4 Discussion

HFMD is a potentially life-threatening illness. Discovering relevant markers for early identification of potentially fatal cases may reduce the mortality rate. We found that sex, OH time, coma, light reflex insensitivity, pulmonary rales, edema or hemorrhage, cold extremities (indicating hypoperfusion), heart rate, hypotension, WBC count, blood glucose concentration, serum lactate level, CK-MB level, and acidosis were all associated with mortality. The results of the multivariate analysis identified female sex, light-reflex insensitivity, higher heart rate, and serum lactate levels as independent risk factors, and longer OH time as an independent protective factor for death. There were no significant differences in age, contact history, peak body temperature, atypical rashes, vomiting, myoclonic jerks, headache, platelet count, ALT level, electrolyte disturbances, and EV71 infection between patients in the survival and non-survival groups. Our results are not in keeping with those of a study from Singapore, which showed that vomiting, the absence of mouth ulcers, atypical presentation, and a raised total WBC count may be associated with mortality.^[8]^ This may be because our study subjects had critical and severe HFMD and because there were more deaths in our study (*n* = 127) than in that study (*n* = 7).

In China, studies showed that life-threatening symptoms and signs such as pulmonary edema, a sudden onset of tachycardia, tachypnea, and cyanosis occurred in HFMD patients between 12 hours to 5 days after the disease onset; thus, early diagnosis and treatment were conducted to reduce mortality.^[10,15,16]^ Our study showed that the average OH time of critical and severe HFMD was 3.2 ± 1.26 days. The shorter the OH time, the faster the disease progressed and the higher risk of death. And a longer OH time was an independent protective factor for death. Therefore, the critical and sever HFMD patients with shorter OH times should be given more attention.

Our data indicate that the female sex may be associated with mortality, but there was no statistical difference between the 2 genders of OH times (male vs female: 3.11 ± 1.31 days vs 3.37 ± 1.16 days, *P* = .17), and there was no interaction effect between the 2 factors (*P* = .07). To the best of our knowledge, no similar finding has been previously reported, either in China or globally. We found no plausible explanation for this finding; thus, more studies are needed to confirm this result.

Our study showed that of the central nervous system symptoms assessed, coma and light-reflex insensitivity were identified as risk factors for death; the latter was an independent risk factor. Thus, evaluating the light reflex is important in monitoring a patient's status.

Almost all previously reported cases of death from critical and severe HFMD were due to pulmonary edema or hemorrhage.^[17,18]^ In Malaysia and Taiwan, fulminant neurogenic pulmonary edema has been reported in patients who died from HFMD.^[6,19,20]^ However, this situation has improved with the accumulation of clinical treatment experience. Damage to certain areas of the brainstem due to encephalitis might cause neurogenic pulmonary edema.^[15,21]^ Several mechanisms have been proposed to explain the pathogenesis of neurogenic pulmonary edema, including an increase in pulmonary vascular pressure and pulmonary endothelial permeability.^[22]^ Our study found that pulmonary edema or hemorrhage, although associated with mortality, were not independent risk factors for death.

Tachycardia was identified as an independent risk factor for fatal cases of HFMD in the present study. Tachycardia can be considered as a sign of autonomic dysfunction from central nervous system complications or it may be a sign of cardiac involvement.^[23]^ A study in China involving 176 children demonstrated that patients with critical and severe HFMD and circulatory collapse often had high blood pressures and heart rates in the early stage.^[13]^ Another study suggested that high levels of catecholamines are released into the peripheral blood due to brainstem encephalitis.^[24]^ These catecholamines excite cardiac β-receptors and peripheral vascular α-receptors, thus, increasing heart rate and blood pressure. If this disorder is not promptly treated, a sudden drop in blood pressure and heart rate can occur within a few hours.^[24–26]^ Secondary hypotension may occur due to catecholamine depletion, affecting circulating blood volume deficiency caused by pulmonary edema or fluid restriction and catecholamine-related cardiac toxicity.^[27]^ In our study, the incidence of hypoperfusion (cyanosis, cold extremities, and hypotension) and tachycardia were higher in the non-survival group than in the survival group. Tachycardia, particularly, was an independent risk factor for death. Therefore, screening children with critical and severe HFMD for these abnormal vital signs is important in predicting impending circulatory failure, allowing the timely initiation of appropriate interventions.

Elevated blood glucose concentration and WBC count generally indicate a poor prognosis.^[28]^ A study conducted in Singapore to determine the risk factors for predicting death in patients with HFMD showed that an elevated WBC count was a risk factor and that physicians should be aware that in such patients the illness may take a fatal course.^[8,29]^ Chang et al reported in their study that patients with HFMD died when their blood glucose concentration increased from 14.1 to 42.2 mmol/L and WBC count increased from 11.6 to 40.6 × 10^9^ cells/L.^[16]^ Lin et al reported that 15 patients with an average blood glucose concentration of 7.66 mmol/L and WBC count of 11.72 × 10^9^ cells/L recovered after treatment, whereas 9 patients with an average blood glucose concentration of 13.84 mmol/L and WBC count of 16.36 × 10^9^ cells/L had adverse sequelae or died.^[30]^ In our study, the values of these 2 variables were also significantly higher in the non-survival group than in the survival group, although they were not identified as independent risk factors for death. Nonetheless, they should be regarded as very helpful in predicting the outcome of a patient with HFMD.

We also found that the serum lactate level differed significantly between the non-survival and survival groups; it was an independent risk factor for death. It has been reported that an increase in the concentration of circulating catecholamines promotes anaerobic glycolysis and vasoconstriction. Such changes induce microcirculatory disturbances and decrease perfusion, resulting in elevated serum lactate levels.^[13]^

Consistent with our findings, some researchers found that a high concentration of serum CK-MB is an independent risk factor for death from HFMD.^[31]^ Although autopsy studies have found no obvious evidence of myocarditis in HFMD,^[24,32]^ significant coagulative myocytolysis, myofibrillar degeneration, and cardiomyocyte apoptosis have been observed.^[24]^ Further research is required to determine whether myocardial damage is a major risk factor for death in patients with HFMD.^[33]^

EV71 is a neuronophagic virus that mainly affects the brainstem, causing encephalitis, aseptic meningitis, and other neurological disorders characterized by vomiting, coma, startle, frequent convulsions, and light-reflex insensitivity. In 2000, a published report demonstrated that a large number of patients with complicated HFMD were infected with EV71.^[34]^ In the present study, EV71 positivity was not associated with HFMD-related death. We considered that EV71 may have been associated with death in the 55 cases in the non-survival group with missing virology investigation data. Moreover, 23 EV71-negative patients also died; these results reiterate that other types of enterovirus infections cannot be ignored.

## 5 Conclusion

Female sex, light-reflex insensitivity, tachycardia, and higher serum lactate levels are likely independent risk factors; and longer OH time is possibly an independent protective factor for death in patients with critical and severe HFMD. Early diagnosis and treatment are beneficial in reducing mortality. The vital signs and Glasgow Coma Scale scores of patients with these risk factors should be carefully monitored. In addition, we propose that in patients with possible central nervous system involvement, fluids should be managed judiciously because of the risk of pulmonary edema and/or hemorrhage.
